# Manipulating Planting Density and Nitrogen Fertilizer Application to Improve Yield and Reduce Environmental Impact in Chinese Maize Production

**DOI:** 10.3389/fpls.2017.01234

**Published:** 2017-07-12

**Authors:** Cailong Xu, Shoubing Huang, Beijing Tian, Jianhong Ren, Qingfeng Meng, Pu Wang

**Affiliations:** ^1^College of Agronomy and Biotechnology, China Agricultural University Beijing, China; ^2^Institute of Crop Sciences, Chinese Academy of Agricultural Sciences Beijing, China

**Keywords:** high yield, high N use efficiency, greenhouse gas intensity, maize, sustainable

## Abstract

Relatively low nitrogen (N) efficiency and heavy environmental costs caused by excessive N fertilizer applications with outdated fertilization techniques are current cultivation production problems with maize among smallholders in North China Plain. Although many studies have examined agronomical strategies for improving yields and N use, the integrated effects of these measures and the associated environmental costs are not well understood. We conducted a 2-year field study with two densities (67,500 plants ha^-1^, which was similar to local farmers’ practices, and 90,000 plants ha^-1^) and three N rates (0, 180, and 360 kg ha^-1^, the rate local farmers’ commonly apply) to test the integrated effects for maize production at Wuqiao experimental station in North China Plain. The higher planting density produced significant increases in grain yield (GY), N use efficiency (NUE), agronomic N efficiency (AEN), and N partial productivity (PFP_N_) by 6.6, 3.9, 24.7, and 8.8%, respectively; in addition, N_2_O emission and greenhouse gas intensity decreased by 7.3 and 4.3%, respectively. With a lower N application rate, from 360 to 180 kg ha^-1^, GY was unchanged, and NUE, AEN, and PFP_N_ all significantly increased by 6.2, 96.0, and 98.7%, respectively; in addition, N_2_O emission and greenhouse gas intensity decreased by 61.5 and 46.2%, respectively. The optimized N rate (180 kg N ha^-1^) for the 90,000 plants ha^-1^ treatment achieved the highest yield with only 50% of the N fertilizer input commonly employed by local farmers’ (360 kg N ha^-1^), which contributed to the increased N-uptake and N-transfer capacity. Therefore, our study demonstrated that agronomical methods such as increasing planting density with reasonable N application could be useful to obtain higher GY along with efficient N management to help lower environmental costs of maize production.

## Introduction

To meet the increased demands of a burgeoning human population for food, feed, fiber, and biofuel, it has been estimated that agricultural production must increase by at least 50%, and perhaps by as much as 110%, relative to production in 2006 (Keyzer et al., 2005; Tester and Langridge, 2010; Tilman et al., 2011). In particular, the production of maize, a globally important crop, must roughly double to meet growing demands (Shiferaw et al., 2011). Nitrogen (N) is important nutrient to maximize crop growth, thus it is often applied to agricultural crops if available (Tilman et al., 2011). Although N fertilizer application can improve maize yields, if overused, it can also have negative environmental impacts such as groundwater pollution through nitrate leaching or increased global warming resulted to N_2_O emissions (Sylvester-Bradley and Kindred, 2009; Burney et al., 2010). Therefore, it is essential to understand the trade-offs between agronomic strategies and N application for crop productivity, nitrogen use efficiency (NUE), and environmental cost.

Winter wheat-summer maize double cropping is the main cropping system in the North China Plain, which accounts for about one third of national maize production. Significant crop production increases have been achieved through improved agronomic and nutrient management with various crop varieties (Grassini et al., 2011; Van Ittersum and Cassman, 2013; Van Ittersum et al., 2013). Planting density, i.e., number of plants per unit area, has proven to be a very effective agronomic strategy to improve maize grain yield (GY) (Tollenaar and Lee, 2002; Ciampitti and Vyn, 2012). For example, in the United States of America, average planting density increased from 30,000 plants ha^-1^ in the 1930s to 45,000 plants ha^-1^ in the 1960s, and average maize yields increased from 2.3 Mg ha^-1^ to 5.5 Mg ha^-1^; with increased planting densities from 55,000 plants ha^-1^ in the 1990s to 97,500 plants ha^-1^ in the 2000s, maize yields increased from 9.0 Mg ha^-1^ to 15.0 Mg ha^-1^ (Zhao and Wang, 2009). However, in China, in the 2000s, the average maize yield was only 5.4 Mg ha^-1^ at a planting density of 60,000 plants ha^-1^ (Li and Wang, 2009). High-yield records in maize production worldwide have been obtained under high planting densities. In United States, the highest maize GY was 33 Mg ha^-1^ with a planting density of 140,790 plants ha^-1^ in Charles City in 2015 (NCGA, 2017). In China, the highest maize GY was 19 Mg ha^-1^ with a planting density of 102,030 plants ha^-1^ in Shandong Province in 2005 (Li and Wang, 2009). Thus, density control as a means to influence yields is an important agronomic method to consider for maize production.

The N fertilizer application has also been used to increase crop yields globally (Miao et al., 2011; Linquist et al., 2013). For United States maize, GY increased from 4.5 to 6.0 Mg ha^-1^ with N application of 30–145 kg ha^-1^ during 1960–1980, while the GY continually increased from 6.0 to 10 Mg ha^-1^ without further N input since 1980, and thus the N partial productivity (PFP_N_) increased by 36%, from 42 kg kg^-1^ in 1980 to 57 kg kg^-1^ in 2000, at the end of 20th century (Cassman et al., 2002). The yield improvements were realized by adopting more efficient technologies and improved N fertilizer management (Pikul et al., 2005; Zhang et al., 2015). However, in China, N fertilizers are typically applied at levels, much higher than the uptake demand of crops, thus, environmental costs (i.e., N leaching/N_2_O emissions) are increased (Zhang et al., 2008; Guo et al., 2010). The main greenhouse gas emitted from agricultural production, N_2_O, is released from soils following the application of N fertilizer; it represents 38% of the total direct greenhouse gas emissions from global agriculture (Burney et al., 2010). The relationship between N_2_O and N fertilizers is usually demonstrated to be exponential (Hoben et al., 2011; Wang et al., 2014). Optimal N management improves crop yields, but it also contributes to higher NUE, thus reducing environmental costs (Chen et al., 2011; Cui et al., 2013). Therefore, it is urgent that we understand the relationship between increasing yields and the management of planting density and N application in an effort to balance agronomical and environmental objectives (e.g., reducing N_2_O emissions or greenhouse gas per unit crop yield) in an environmentally sustainable manner.

Some studies have reported that high maize GY through high NUE and relatively low N application were achieved under close planting because of high biomass or N accumulation and allocation to grain (Cui et al., 2009; Ciampitti et al., 2013). Moll et al. (1982) determined that N utilization can be divided into two processes: N-uptake efficiency and N-transfer efficiency. N-uptake is a reflection of the capacity of the plant to recover N from fertilizer and soil (Moll et al., 1982; Foulkes et al., 2009) depending on the amount of root length density and the uninterrupted carbohydrate mobilization from shoot to root (Tolley-Henry et al., 1988; Rajcan and Tollenaar, 1999). The N-transfer efficiency is the ability of the plant to transfer the N taken up by the crop into the grain during the grain-filling period (Moll et al., 1982; Foulkes et al., 2009). The vegetative organs, particularly the green leaf tissue, were the major storage organs for N, 49–53% of the total N accumulation at silking of maize, and the source of N for grain filling (Liu et al., 2014). Previous studies demonstrated that the absorption and remobilization of N in plants were both affected by planting density and N management (Ciampitti and Vyn, 2013; Kosgey et al., 2013). A more thorough study of planting and N management interactions is necessary to understand N-uptake and N-transfer responses and their relationship to final N utilization within maize plants.

The aim of this study was to examine the effect of planting density and N application rate on GY, N utilization, N_2_O emission intensity, and greenhouse gas intensity of summer maize in 2 years under field conditions. We also investigated the N accumulation and N remobilization of summer maize to elucidate the processes involved in increasing N utilization by optimizing planting density and N application rate.

## Materials and Methods

### Site Description

**FIGURE 1:**
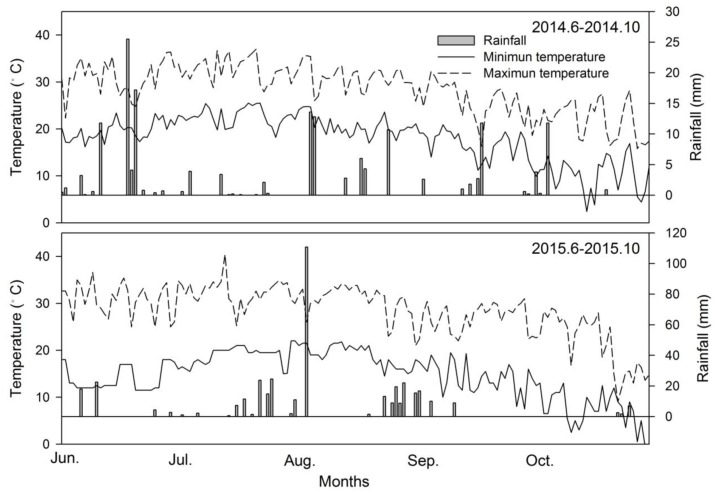
Daily temperature and rainfall during the maize growing season in 2014 and 2015.

Field experiments were carried out in 2014 and 2015 at the Wuqiao Experiment Station of China Agricultural University, Hebei province, China (37° 41′ N, 116° 36′ E). In the upper 0.4 m of the clay-loam soils, pH was 8.3 with a bulk density of 1.45 g cm^-3^, and they contained 12.36 g kg^-1^ of soil organic matter, 1.04 g kg^-1^ of total nitrogen (N), 37.71 mg kg^-1^ of readily available phosphorous (P), and 94.22 mg kg^-1^ of readily available potassium (K). The soil pH, organic matter, total N, available P and K were analyzed by following procedures of Piper (1950), Bremner (1965), Kjeldahl method, Stanford and English (1949) and Olsen et al. (1954), respectively. Preceding crop was winter wheat. A standard agro-meteorological station automatically recorded meteorological conditions in the experimental fields; air temperature and rainfall data during the study period growing seasons are provided in **Figure 1**. The light and temperature condition in 2015 was better than that in 2014, which benefited maize production.

### Experimental Design and Treatments

The experimental design was a split-plot with three replicates: two planting densities treatments were applied as the main plots, and three N treatments were designated as sub-plots. The size of the main plot was 20 m × 15 m, and the sub-plot size was 20 m × 5 m. A common maize hybrid – Zhengdan 958, the main maize cultivar in NCP, was planted at two planting densities (67,500 plants ha^-1^ and 90,000 plants ha^-1^) with equal row spacing of 0.6 m. The N application rates were as follows: control (N0), 180 kg N ha^-1^ (N180), and 360 kg N ha^-1^ (N360). As recommended by Liu et al. (2003), the N180 treatment was set in two stages: 90 kg N ha^-1^ at the three-leaf stage and 90 kg N ha^-1^ at the silking stage. The N360 treatment was set at 360 kg N ha^-1^ at the time of planting; this is the traditional N management protocol used by farmers in NCP (Zhang et al., 2008). Urea was applied 0.1 m deep into the soil with a furrowing machine according to the N application rates. In addition, all plots received 130 kg P_2_O_5_ ha^-1^ in the form of calcium superphosphate (P_2_O_5_ 12%) and 130 kg K_2_O ha^-1^ in the form of potassium sulfate (K_2_O 60%) at the time of planting. Maize was planted on June 15, 2014 and on June 19, 2015, after winter wheat crops were harvested. Each plot was irrigated with 75 mm of water immediately after sowing. The maize grains were harvested on October 5 in both years before winter wheat planting.

### Plant Sampling and Analysis

Three plant samples were randomly selected from the center of each plot during the silking stage and at the time of harvest. Plants were dissected into leaf + husk, stalk + cob, and grain (only at the time of harvest). All separated components were oven-dried at 80°C to a constant weight; they were then weighed to record dry matter accumulation (DMA, kg ha^-1^) and milled into a powder. Total N was measured using the Kjeldahl method, and NUE (kg kg^-1^), agronomic N efficiency (AEN, kg kg^-1^), and N partial productivity (PFP_N_, kg kg^-1^) were calculated using the methods of Dobermann (2005).

$$\text{NUE}=\frac{\text{grain yield}}{\text{total N uptake by plant}}$$

$$\text{AEN}=\frac{\text{grain yield with applied N-grain yield without N}}{\text{N application amount}}$$

$${\text{PFP}}_{\text{N}}=\frac{\text{grain yield}}{\text{N application amount}}$$

Based on the DMA and N accumulation measurements, we calculated the following parameters (Mi et al., 2003; Chen Y. et al., 2014):

$$\text{Post silking DMA }(\text{kg }{\text{ha}}^{-1})=\text{DMA at harvest-DMA at silking}$$

$$\begin{array}{c} N\,\;remobilazation\,\;from\,\;vegetative\,\;organs\,\;to\,\;grain\,\;(kg\,\;{ha}^{-1})\\ =\;N\,\;accumulation\,\;in\,\;vegetative\,\;organs\,\;at\,\;silking\\ -N\,\;accumulation\,\;in\,\;vegetative\,\;organs\,\;at\,\;harvest \end{array}$$

$$\text{N remobilization efficiency }(\%)=\frac{\text{N remobilization from vegetative organs to grain}}{\text{N accumulation in vegetative organs at silking}}$$

To determine the green leaf area at the silking stage and 10, 20, 30, 40, and 50 days after silking (DAS), leaf length (L, cm), and maximum width (W, cm) were measured to calculate green leaf area (Montgomery, 1911) and leaf area index (LAI).

$$\text{Green leaf area }({\text{cm}}^{2})=0.75\times \text{L}\times \text{W}$$

$$\text{Leaf area index=}\frac{\text{total green area per plant}}{\text{land area per plant}}$$

Direct N_2_O emission (kg N ha^-1^), NH_3_ volatilization (kg N ha^-1^), and NO_3_^-^ leaching (kg N ha^-1^) were calculated using the methods of Cui et al. (2013).

$$\text{Direct N}{}_{2}\text{O emission}={0.48e}^{0.0058X}$$

$${\text{NH}}_{3}\,\text{volatilization}=0.24X+1.3$$

$${\text{NO}}_{3}-\text{leaching=}{4.46e}^{0.0094X}$$

X is the N application rate (kg N ha^-1^). The indirect N_2_O emissions can be estimated by following the IPCC methodology (IPCC, 2006) where 1 and 0.75% of the volatilized N-NH_3_ and leached N-NO_3_ is lost as N_2_O-N. Using the above N loss-N input response curve, we calculated the direct, indirect, and total N_2_O emissions (kg N ha^-1^), and N_2_O emission intensity (N_2_O emission per unit maize yield, kg N Mg^-1^).

Total greenhouse gas emissions (kg CO_2_ eq; including CO_2_, CH_4_, and N_2_O) during the life cycle of maize production are represented in three components: emissions during N fertilizer application; emissions during N fertilizer production and transportation; and emissions during the production and transportation of P and K fertilizer, and pesticides, and diesel fuel use in farming operations such as sowing, tilling, and harvesting. Components were calculated according to the methods of Cui et al. (2013).

$$\text{Greenhouse gas emission during N use=298}\times {\text{N}}_{2}{\text{O}}_{\text{total}}\times 44÷28$$

$$\text{Greenhouse gas emission during N production=}{\text{N}}_{\text{input}}\times 8.21$$

$$\begin{array}{c} Greenhouse\,\;gas\,\;emission\,\;during\,\;others\,\;pathway\\ =P_{2}O_{5input}\times {EF}_{p}+K_{2}O_{input}\times {EF}_{k}+{Pest}_{.input}\\ \times {EF}_{pest.}+{Fuel}_{.input}\times {EF}_{fuel.}+9.2\times Irri.\times {EF}_{elec.} \end{array}$$

EF in the equation 14 is coefficient of greenhouse gas emissions. The values of EF_p_, EF_k_, EF_pest._, EF_fuel._, and EF_elec._ were 0.79, 0.55, 19.13, 3.75, and 1.14 kg CO_2_ eq per unit input, respectively. The value of Pest_.input_ and Fuel_.input_ were estimated to be 4.13 and 72.7 kg ha^-1^, respectively. We then calculated the total greenhouse gas emission (also known as global warming potential, kg CO_2_ eq ha^-1^) and greenhouse gas intensity (kg CO_2_ eq Mg^-1^).

At the time of harvest, maize GY (14% water content) was measured within a randomly selected 7.2 m^2^ sub-plot (4 m × 1.8 m) in each plot. Kernel number was measured on 10 randomly selected ears from each plot. Thousand-kernel weight (TKW) was determined after drying thousand-kernel samples at 80°C in a forced-draft convention oven to a constant weight.

### Data Analysis

The effects of the treatments and years on the measured parameters [including GY, N utilization, N_2_O emission intensity, and greenhouse gas intensity] were evaluated using univariate analysis of variance (ANOVA) procedures. After verifying the homogeneity of error variances, all the data across planting densities and N application rates were pooled for use in the ANOVA. Differences were compared using the least significant difference test (LSD) at a 0.05 level of probability. All analyses were conducted using SPSS 17.0 (SPSS Inc., Chicago, IL, United States).

## Results

### Grain Yield and Yield Components

Grain yields and yield components were significantly affected by planting density, N application rate, and the interaction of planting density × N application rate (**Table 1** and **Supplementary Figure S1**). As planting density increased from 67,500 to 90,000 plants ha^-1^, GY was significantly increased by 7% (from 9,556.5 to 10,184.5 kg ha^-1^) across years and N application rates. No significant difference in GY was observed between N180 and N360 treatments across years and two densities (**Table 1**).

**Table 1:** Grain yield and yield components of summer maize for planting densities of 67,500 (D67500) and 90,000 plants ha^-1^ (D90000) and N treatments of 0 (N0), 180 (N180), and 360 kg N ha^-1^ (N360) in 2014 and 2015.

Treatments	Kernels (No. ear^-1^)	TKW (g)	Ear(No. ha^-1^)	Grain yield (kg ha^-1^)
**Year**				
2014	474.7a^†^	283.0b	75930.2a	9646.7a
2015	476.4a	299.3a	76774.7a	10094.2a
**Density (plants ha^-1^)**				
D67500	492.0a	296.9a	68371.6b	9556.5b
D90000	459.0b	285.4b	84333.3a	10184.5a
**Nitrogen (kg N ha^-1^)**				
N0	417.2b	286.9b	74714.8b	6879.4b
N180	507.6a	290.6ab	77213.0a	11331.5a
N360	501.8a	295.9a	77129.6a	11400.6a
**Source of variation**				
Year (Y)	NS	^∗∗∗^	NS	^∗^
Density (D)	^∗∗∗^	^∗∗∗^	^∗∗∗^	^∗∗^
Nitrogen (N)	^∗∗∗^	^∗∗∗^	^∗∗^	^∗∗∗^
Y × D	NS	NS	NS	NS
Y × N	NS	NS	NS	NS
D × N	NS	NS	^∗∗^	^∗^
Y × D × N	NS	NS	NS	NS


*^†^Different letters within year, density or nitrogen indicate significant differences (*P* < 0.05). NS, no significant (*P* > 0.05). TKW, thousand-kernel weight. ^∗^Significant at *P* < 0.05. ^∗∗^Significant at *P* < 0.01. ^∗∗∗^Significant at *P* < 0.001.*

Increased planting density produced a significant 23% increase of average ear number; kernel number and TKW displayed significant decreases of 7 and 4%, respectively (**Table 1**). No significant differences in the three yield components were observed between N180 and N360 treatments.

### Nitrogen Utilization

Planting density, N application rate, and the interaction of planting density × N application rate had a significantly influence on NUE, AEN and PFP_N_ (**Table 2** and **Supplementary Figure S2**). As planting density increased from 67,500 to 90,000 plants ha^-1^, NUE, AEN, and PFP_N_ significantly increased by 4, 25, and 9%, respectively. Across years and planting densities, the average NUE was ranked in the following order: 44.4 kg kg^-1^ for N180 > 41.8 kg kg^-1^ for N360 > 32.5 kg kg^-1^ for N0. The AEN and PFP_N_ in N180 were 24.7 and 63.0 kg kg^-1^, respectively, which displayed significant increases of 96 and 99%, respectively, compared to N360 (**Table 2**).

**Table 2:** Nitrogen use efficiency (NUE), agronomic N efficiency (AEN), and N partial factor productivity (PFP_N_) of summer maize for planting densities of 67,500 (D67500) and 90,000 plants ha^-1^ (D90000) and N treatments of 0 (N0), 180 (N180), and 360 kg N ha^-1^ (N360) in 2014 and 2015.

Treatments	NUE (kg kg^-1^)	AEN (kg kg^-1^)	PFP_N_ (kg kg^-1^)
**Year**			
2014	37.6b^†^	17.9a	46.1a
2015	41.6a	19.4a	48.5a
**Density (plants ha^-1^)**			
D67500	38.8b	16.6b	45.3b
D90000	40.3a	20.7a	49.3a
**Nitrogen (kg N ha^-1^)**			
N0	32.5c		
N180	44.4a	24.7a	63.0a
N360	41.8b	12.6b	31.7b
**Source of variation**			
Year (Y)	^∗∗∗^	NS	NS
Density (D)	^∗∗^	^∗∗∗^	^∗∗∗^
Nitrogen (N)	^∗∗∗^	^∗∗∗^	^∗∗∗^
Y × D	NS	NS	NS
Y × N	NS	NS	NS
D × N	^∗∗∗^	NS	NS
Y × D × N	NS	NS	NS


*^†^Different letters within year, density or nitrogen indicate significant differences (*P* < 0.05). NS, no significant (*P* > 0.05). ^∗∗^Significant at *P* < 0.01. ^∗∗∗^Significant at *P* < 0.001.*

### Nitrogen Accumulation

Planting density and N application rate had a significant influence on N accumulation at silking and harvest, and the N distribution ratio at the time of harvest (**Tables 3**, **4**).

**Table 3:** Nitrogen accumulation and distribution ratio at harvest of summer maize for planting densities of 67,500 (D67500) and 90,000 plants ha^-1^ (D90000) and N treatments of 0 (N0), 180 (N180), and 360 kg N ha^-1^ (N360) in 2014 and 2015.

Treatments	N accumulation (kg ha^-1^)				N distribution ratio (%)		
		
	Grain	Leaf + husk	Stalk + cob	Total	Grain	Leaf + husk	Stalk + cob
**Year**							
2014	163.6a^†^	47.5a	44.9a	254.5a	64.4a	18.6a	17.6a
2015	152.2b	44.2b	42.2b	238.5b	63.9b	18.5a	17.7a
**Density (plants ha^-1^)**							
D67500	152.8b	46.8a	44.6a	243.4b	62.8b	19.2a	18.3a
D90000	163.0a	44.8b	42.5b	249.6a	65.5a	17.8b	17.0b
**Nitrogen (kg N ha^-1^)**							
N0	138.7c	37.2c	36.1c	211.3c	65.6a	17.7c	17.1b
N180	163.0b	46.6b	45.0b	254.6b	64.0b	18.3b	17.7a
N360	172.0a	53.7a	49.5a	273.7a	62.8c	19.6a	18.1a
**Source of variation**							
Year (Y)	^∗∗∗^	^∗∗∗^	^∗∗∗^	^∗∗∗^	^∗∗^	NS	NS
Density (D)	^∗∗∗^	^∗∗∗^	^∗∗∗^	^∗∗∗^	^∗∗∗^	^∗∗∗^	^∗∗∗^
Nitrogen (N)	^∗∗∗^	^∗∗∗^	^∗∗∗^	^∗∗∗^	^∗∗∗^	^∗∗∗^	^∗∗∗^
Y × D	^∗∗^	NS	NS	^∗∗^	NS	NS	NS
Y × N	^∗∗∗^	^∗^	^∗^	^∗∗∗^	NS	NS	NS
D × N	^∗∗∗^	^∗∗∗^	^∗^	^∗∗∗^	^∗∗∗^	^∗∗∗^	NS
Y × D × N	^∗∗^	^∗^	^∗∗^	^∗∗∗^	NS	NS	NS


*^†^Different letters within year, density or nitrogen indicate significant differences (*P* < 0.05). NS, no significant (*P* > 0.05). ^∗^Significant at *P* < 0.05. ^∗∗^Significant at *P* < 0.01. ^∗∗∗^Significant at *P* < 0.001.*

**Table 4:** Nitrogen accumulation of vegetative organs at silking, and its remobilization and remobilization efficiency to grain after silking of summer maize for planting densities of 67,500 (D67500) and 90,000 plants ha^-1^ (D90000) and N treatments of 0 (N0), 180 (N180), and 360 kg N ha^-1^ (N360) in 2014 and 2015.

Treatments	N accumulation (kg ha^-1^)			N remobilization (kg ha^-1^)			N remobilization Efficiency (%)		
			
	Leaf + husk	Stalk + cob	Total	Leaf + husk	Stalk + cob	Total	Leaf + husk	Stalk + cob	Total
**Year**									
2014	85.3b^†^	64.6b	150.0b	41.1b	22.5b	63.5b	48.4b	34.7b	42.5b
2015	97.0a	71.5a	168.5a	49.5a	26.6a	76.2a	51.1a	36.9a	45.1a
**Density (plants ha^-1^)**									
D67500	87.8b	63.2b	151.0b	41.0b	18.6b	59.6b	46.8b	29.7b	39.6b
D90000	94.5a	73.0a	167.4a	49.6a	30.4a	80.1a	52.7a	41.9a	48.0a
**Nitrogen (kg N ha^-1^)**									
N0	83.3c	63.5c	146.8c	42.5b	22.8b	65.3b	51.0a	39.5a	45.5a
N180	89.0b	67.8b	157.0b	47.4a	25.4a	72.8a	53.3a	37.5a	43.4ab
N360	101.1a	72.9a	174.0a	48.1a	25.9a	74.0a	47.6b	35.5b	42.5b
**Source of variation**									
Year (Y)	^∗∗∗^	^∗∗∗^	^∗∗∗^	^∗∗∗^	^∗∗∗^	^∗∗∗^	^∗∗∗^	^∗∗∗^	^∗∗∗^
Density (D)	^∗∗∗^	^∗∗∗^	^∗∗∗^	^∗∗∗^	^∗∗∗^	^∗∗∗^	^∗∗∗^	^∗∗∗^	^∗∗∗^
Nitrogen (N)	^∗∗∗^	^∗∗∗^	^∗∗∗^	^∗∗∗^	^∗∗∗^	^∗∗∗^	^∗∗∗^	^∗∗∗^	^∗∗∗^
Y × D	NS	^∗∗^	^∗∗^	^∗^	^∗∗^	^∗∗^	NS	NS	NS
Y × N	NS	^∗∗^	^∗∗^	NS	^∗^	NS	NS	NS	NS
D × N	^∗∗^	^∗∗∗^	^∗∗∗^	^∗^	^∗∗∗^	^∗∗^	^∗∗^	^∗∗∗^	NS
Y × D × N	NS	^∗^	NS	NS	NS	NS	NS	NS	NS


*^†^Different letters within year, density or nitrogen indicate significant differences (*P* < 0.05). NS, no significant (*P* > 0.05). ^∗^Significant at *P* < 0.05. ^∗∗^Significant at *P* < 0.01. ^∗∗∗^Significant at *P* < 0.001.*

As planting density increased from 67,500 to 90,000 plants ha^-1^, N accumulation at silking and harvest of summer maize were significantly increased by 11 and 3%, respectively, across years and N applying rates. At silking, a planting density of 90,000 plants ha^-1^ displayed increases in N accumulation in leaf + husk and stalk + cob of 8 and 16%, respectively, relative to a summer maize planted at a density of 67,500 plants ha^-1^. At harvest, the higher planting density resulted in significantly increased N accumulation in grains, however, the N accumulation in leaf + husk and stalk + cob were decreased by 4 and 5%, respectively (**Tables 3**, **4**).

Across years and planting densities, we observed increased total N accumulation at silking, N accumulation in each organ at silking, total N accumulation at harvest, and N accumulation in each organ at harvest with increasing N application rates (**Tables 3**, **4**).

### Nitrogen Remobilization

Planting density, N application rate, and the interaction of planting density × N application rate significantly influenced N remobilization and N remobilization efficiency from vegetative organ to grain after silking (**Table 4**).

N remobilization of total, leaf + husk, and stalk + cob was increased by 34, 21, and 39% as planting density increased from 67,500 to 90,000 plants ha^-1^, respectively; in addition, N remobilization efficiency of total, leaf + husk, and stalk + cob was also increased by 21, 13, and 41%, respectively (**Table 4**). No significant differences in N remobilization of total, leaf + husk, and stalk + cob were observed between N180 and N360 treatments. However, N remobilization efficiency of total, leaf + husk, and stalk + cob in N360 treatment was decreased by 2, 11, and 5% relative to summer maize of N180 treatment (**Table 4**).

### Dry Matter Accumulation and Leaf Area Index

Pre-silking, post-silking, and total DMA were significantly affected by planting densities and N application rates (**Table 5** and **Supplementary Figure S3**).

**Table 5:** Pre- and post-silking dry matter accumulation of summer maize for planting densities of 67,500 (67500) and 90,000 plants ha^-1^ (D90000) and N treatments of 0 (N0), 180 (N180), and 360 kg N ha^-1^ (N360) in 2014 and 2015.

Treatments	Dry matter accumulation (kg ha^-1^)		
	
	Pre-silking	Post-silking	Total
**Year**			
2014	7676.5a^†^	12466.9a	20143.4a
2015	7543.4a	12432.3a	19975.7a
**Density (plants ha^-1^)**			
D67500	7222.5b	12225.1b	19447.6b
D90000	7997.4a	12674.1a	20671.5a
**Nitrogen (kg N ha^-1^)**			
N0	7070.5b	11194.2b	18264.7b
N180	7490.4ab	13228.4a	20718.9a
N360	8268.9a	12926.2a	21195.1a
**Source of variation**			
Year (Y)	NS	NS	NS
Density (D)	^∗^	^∗∗^	^∗∗^
Nitrogen (N)	^∗^	^∗∗∗^	^∗∗∗^
Y × D	NS	NS	NS
Y × N	NS	NS	^∗∗^
D × N	^∗^	^∗∗∗^	NS
Y × D × N	NS	^∗^	^∗^


*^†^Different letters within year, density or nitrogen indicate significant differences (*P* < 0.05). NS, no significant (*P* > 0.05). ^∗^Significant at *P* < 0.05. ^∗∗^Significant at *P* < 0.01. ^∗∗∗^Significant at *P* < 0.001.*

**FIGURE 2:**
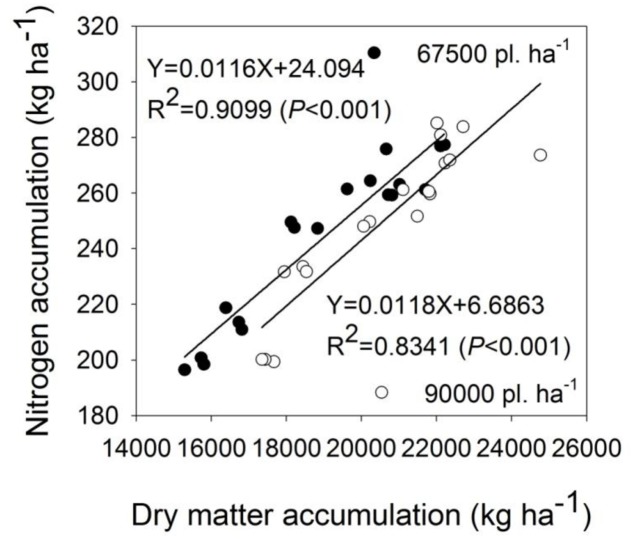
The relationship between nitrogen accumulation and dry matter accumulation at harvest of summer maize in the planting densities of 67,500 and 90,000 kg ha^-1^.

With the higher planting density, the pre-silking, post-silking, and total DMA were significantly increased by 11, 4, and 6%, respectively, across years and N application rates. The post-silking and total DMA were significantly increased by N application, but we observed no significant differences in pre-silking, post-silking, and total DMA when comparing N180 and N360 treatments (**Table 5**). In addition, significant positive correlations were observed between N accumulation and DMA at harvest in both planting densities (**Figure 2**).

**FIGURE 3:**
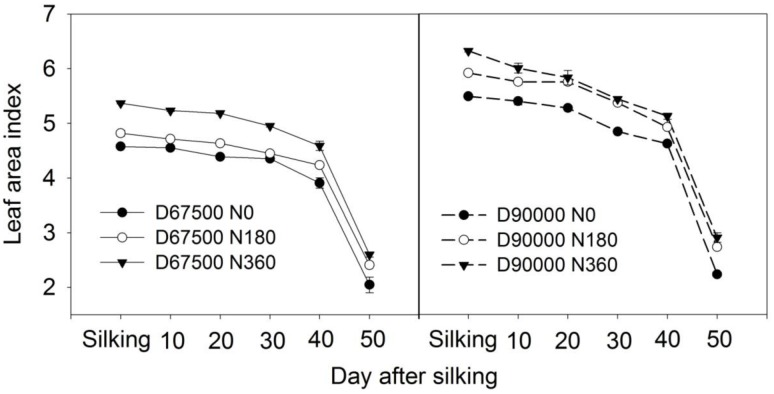
Leaf area index of summer maize after silking for planting densities of 67,500 and 90,000 plants ha^-1^ and N treatments of 0, 180, and 360 kg N ha^-1^ in 2014 and 2015. D67500 and D90000 indicated 67,500 and 90,000 plants ha^-1^, respectively. N0, N180, and N360 indicated 0, 180, and 360 kg N ha^-1^, respectively.

**FIGURE 4:**
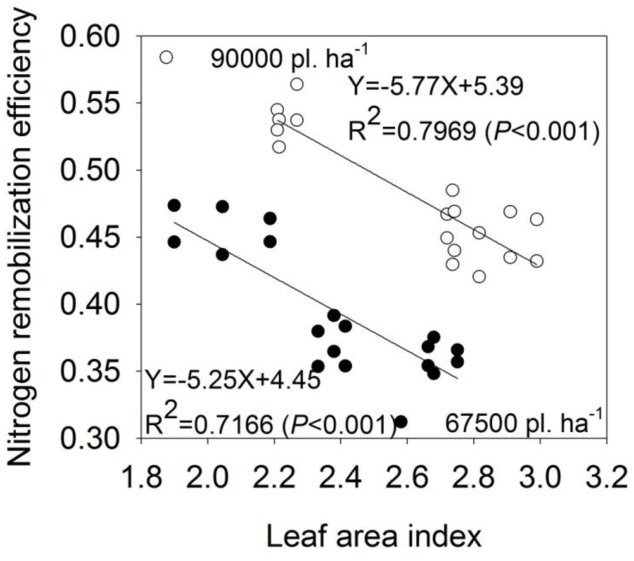
The relationship between nitrogen remobilization efficiency and leaf area index at harvest of summer maize in the planting densities of 67,500 and 90,000 kg ha^-1^.

The LAI of maize gradually decreased after silking. The LAI after silking in N360 treatment was always higher than that in N180 treatment under both planting densities. Given the same N application rate, the LAI in planting density of 90,000 plants ha^-1^ was always higher (**Figure 3**). In addition, significant negative correlations were observed between N remobilization efficiency and LAI at harvest in both planting densities (**Figure 4**).

### N_2_O Emission Intensity and Greenhouse Gas Intensity

N_2_O emission intensity and greenhouse gas intensity were both strongly affected by planting density, N application rate, and the interaction of planting density × N application rate (**Table 6** and **Supplementary Figure S4**).

**Table 6:** N_2_O emission intensity and greenhouse gas intensity of summer maize for planting densities of 67,500 (D67500) and 90,000 plants ha^-1^ (D90000) and N treatments of 0 (N0), 180 (N180), and 360 kg N ha^-1^ (N360) in 2014 and 2015.

Treatments	N_2_O emission intensity (kg N Mg^-1^)			Greenhouse gas intensity (kg CO_2_ eq Mg^-1^)
		
	Direct	Indirect	Total	
**Year**				
2014	0.181a^†^	0.077a	0.258a	398.04a
2015	0.173b	0.074b	0.247b	380.92b
**Density (plants ha^-1^)**				
D67500	0.183a	0.078a	0.262a	402.16a
D90000	0.171b	0.072b	0.243b	376.82b
**Nitrogen (kg N ha^-1^)**				
N0	0.070c	0.070c	0.077c	227.00c
N180	0.121b	0.055b	0.176b	329.43b
N360	0.341a	0.164a	0.505a	612.04a
**Source of variation**				
Year (Y)	^∗^	^∗^	^∗^	^∗^
Density (D)	^∗∗∗^	^∗∗^	^∗∗^	^∗∗^
Nitrogen (N)	^∗∗∗^	^∗∗∗^	^∗∗∗^	^∗∗∗^
Y × D	NS	NS	NS	NS
Y × N	NS	NS	NS	NS
D × N	^∗∗^	^∗∗^	^∗∗^	^∗^
Y × D × N	NS	NS	NS	NS


*^†^Different letters within year, density or nitrogen indicate significant differences (*P* < 0.05). NS, no significant (*P* > 0.05). ^∗^Significant at *P* < 0.05. ^∗∗^Significant at *P* < 0.01. ^∗∗∗^Significant at *P* < 0.001.*

As planting density increased from 67,500 to 90,000 plants ha^-1^, the direct, indirect, and total N_2_O emission intensity were significantly decreased by 7, 8, and 7%, respectively, across years and N application rates. The direct, indirect, and total N_2_O emission intensity were significantly increased by N application. The direct, indirect, and total N_2_O emission intensity in N360 treatments were significantly increased by 182, 198, and 187%, respectively, relative to the N180 treatment (**Table 6**). The higher planting density also produced significantly lower (4%) greenhouse gas intensity across years and N application rates. Greenhouse gas intensity significantly increased with N application; we observed a significant increase of 86% in greenhouse gas intensity in the N360 treatment compared to the N180 treatment (**Table 6**).

## Discussion

### Effects of Planting Density and Nitrogen Application Rate on Grain Yield

Selecting high-yield maize varieties that are tolerant of high planting densities has become an increasingly popular field of research for breeders (Troyer and Rosenbrook, 1983; Tollenaar and Lee, 2002). Previous studies have reported that increases in maize yields are mainly dependent on the breeding of high-yield varieties and high planting densities (Tokatlidis et al., 2011; Van Ittersum and Cassman, 2013). In this study, GY was significantly higher at a planting density of 90,000 plants ha^-1^ (**Table 1** and **Supplementary Figure S1**), which is much higher than the planting densities typically employed by local farmers (<60,000 plants ha^-1^; Chen et al., 2009) and more comparable to planting densities commonly used in North America (>80,000 plants ha^-1^; Lee and Tollenaar, 2007). However, the kernel number and TKW were decreased (**Table 1**), which was in agreement with most previous studies (Andrade et al., 2002; Borrás et al., 2004). Under high planting density, interplant competition for resources is exacerbated, which produces a lower number of kernel per ear and lower TKW (Tollenaar et al., 2006; Boomsma et al., 2009); GY increases are attributable to the increased number of ear per area (Grassini et al., 2011; Van Ittersum and Cassman, 2013). Thus, for GY, the positive effects of high planting densities surpassed the negative effects of interplant competition. Improving kernels per ear and TKW are two potential paths to increase maize yields in high planting density conditions in the future.

Nitrogen can affect crop yields through its influence on the yield components (kernel per ear, TKW, and ear per unit area; Raun and Jhonson, 1999; Van Ittersum and Cassman, 2013). In this study, the GY and yield components were significantly increased by the application of N (N180 and N360 kg N ha^-1^); however, we observed no significant differences in maize GY and yield components between N180 and N360 kg N ha^-1^ treatments (**Table 1**). These observations are consistent with previous studies demonstrating that the response of maize GY to increasing N apply followed a parabolic curvilinear relationship (Cui et al., 2009; Meng et al., 2013). Previous studies have demonstrated that the metabolism of N and carbon in plants are affected by the activity of nitrate reductase (NR) and sucrose phosphate synthetase (SPS; Lawlor and Cornic, 2002; Shen et al., 2007). The activity of these two enzymes shows an increasing trend with increased N applications within a certain range, thus, they enhance the accumulation of photoproduct and transshipment; however, the activity of SPS decreases when the N is excessively applied, which can explain observed decreases in the number of kernel per ear and TKW (Shen et al., 2007; Li et al., 2012), leading to low GY (**Table 1** and **Supplementary Figure S1**).

### Effects of Planting Density and Nitrogen Application Rate on Nitrogen Utilization

Nitrogen utilization can be increased by better-integrated agronomic management practices, such as fertilizing and crop cultivation techniques, which can better ensure maximized crop production and N efficiency (Chen et al., 2006; Cui et al., 2009). Our results demonstrated that high NUE, AEN, and PFP_N_ are obtainable through increased planting density (from 67,500 to 90,000 plants ha^-1^) or decreased N applications (from 360 to 180 kg N ha^-1^; **Table 2** and **Supplementary Figure S2**). Using the same N application rate, the highest NUE, AEN, and PFP_N_ values were obtained with a planting density of 90,000 plants ha^-1^; using the same planting density, the highest NUE, AEN, and PFP_N_ values were both obtained in the N180 treatment. The highest NUE, AEN, and PFP_N_ values among the six treatments were obtained at a planting density of 90,000 plants ha^-1^ with an N application rate of 180 kg N ha^-1^ (**Supplementary Figure S2**), which is comparable to the recommended high-yield maize in China (100,000 plants ha^-1^ with 237 kg N ha^-1^; Chen et al., 2011) and in Nebraska, United States (75,000 plants ha^-1^ with 183 kg N ha^-1^; Grassini et al., 2011).

For N absorption, the correlation analysis indicated that N accumulation had a significant and positive relationship with DMA (**Figure 2**). This is consistent with previous studies that demonstrated that N-uptake and DMA improved simultaneously (Liu et al., 2014). In this study, DMA was greater with higher planting density and N application rates (**Table 5**). Interplant competition at high density contributes to reduced DMA per-plant (Tollenaar et al., 2006; Boomsma et al., 2009). However, maize biomass production at 90,000 plants ha^-1^ treatment increased under field level in the present study (**Table 3**), which is consistent with previous results of Lee and Tollenaar (2007) who found that higher biomass accumulations were obtained at relatively higher densities (75,000–90,000 plants ha^-1^), thus, a high rate of N-uptake was observed in the positive relation between N accumulation and DMA. In addition, higher N concentration and accumulation were mainly due to higher N application rates (Liu et al., 2014). Not surprisingly, higher N accumulation was observed with a planting density of 90,000 plants ha^-1^ and the N360 treatments in this study.

Regarding N-transfer, we observed a decreased efficiency in N remobilization from vegetative organs to grains with increased rates of N, and N remobilization and N remobilization efficiency improved under the higher planting density of summer maize (**Table 4**). Previous researchers indicated that 33–65% of N concentrated in grains comes from N remobilization that was stored in vegetative organs before silking (Ciampitti and Vyn, 2013; Kosgey et al., 2013), especially the leaves, which contributed up to 45–65% (Chen Y. et al., 2014); N remobilization and N efficiency in our study were within those ranges, respectively (**Table 4**). Previous studies showed that high N application rates (e.g., 300 and 400 kg N ha^-1^) contributed to the high N uptake capacity but low N remobilization efficiency because the leaves always maintained a “stay-green” state (Lee and Tollenaar, 2007; Ciampitti and Vyn, 2012). The negative correlation between N remobilization efficiency and LAI in this study reinforces the above conclusion (**Figure 4**). However, under relatively high planting densities (e.g., 90,000 plants ha^-1^ in this study), the N application for each plant was relatively low, and thus the N was not available in excess for each plant.

### Effects of Planting Density and Nitrogen Application Rate on N_2_O Emission Intensity and Greenhouse Gas Intensity

Global N fertilizer consumption is expected to reach 250 Mt yr^-1^ by 2050 (Tilman et al., 2011), which will lead to massive releases of greenhouse gas, water pollution, and other major environmental problems (Tilman et al., 2011; Lu and Tian, 2013). The future of agricultural production not only needs sustainable yield increases, but it must also limit environmental harm caused by excessive N application (Cui et al., 2013; Chen X. et al., 2014). We observed total N_2_O emissions of was 1.99 and 5.74 kg N ha^-1^ in the recommended N management practice (N180; Liu et al., 2003) and the traditional N dose (N360) treatments, respectively; this also confirmed that excessive N fertilizer applications can lead to substantial fertilizer N losses with N entering ecosystems through nitrification-denitrification processes (Robertson and Vitousek, 2009; Sutton et al., 2011). The emission factor was 1.10% in N180 in our study, which was comparable to the 1.20% estimated by Cui et al. (2013) and 1.06% estimated by Linquist et al. (2012). The N180 treatment achieved similar GY with a 62% reduction in N_2_O emission intensity and a 46% reduction in greenhouse gas intensity compared to the N360 treatment (**Table 6**); we attributed this result to competitive GY. In addition, the 7% reduction in N_2_O emission intensity and a 4% reduction in greenhouse gas intensity in crops at a planting density of 90,000 plants ha^-1^ compared to 67,500 plants ha^-1^ were also attributed to the improved crop yields achieved through higher planting densities.

In the present study, compared with farmer practice, the labor costs in comprehensive management practice (increasing planting density and reducing N application) was increased, however, the N input was decreased with higher GY. In brief, the annual profit in comprehensive management practice increased by ¥ 503 per ha compared with farmer practice. In actual maize production, field demonstrations of the revised fertilizer regime could be used to validate the research findings and be used to train farmers to ensure uptake of the new recommendations of best practice.

## Conclusion

Optimization of planting density (90,000 plants ha^-1^) and N application rate (180 kg N ha^-1^) resulted in the highest N utilization (NUE, AEN, and PFP_N_) and GY; it also lowered N_2_O emission intensity and greenhouse gas intensity of summer maize. The increase in N utilization was essentially due to the increased N-uptake capacity and N-transfer capacity. Therefore, higher planting densities and reduced N application rates should be considered to promote improved N utilization and GY with lower environmental costs in maize production.

## Author Contributions

PW and QM designed the study; CX, BT, and JR performed the study; CX, SH, and QM analyzed data and performed the statistical analyses; CX and QM wrote the paper.

## Conflict of Interest Statement

The authors declare that the research was conducted in the absence of any commercial or financial relationships that could be construed as a potential conflict of interest.

## Supplementary Material

**FIGURE S1 |** Grain yield of summer maize for planting densities of 67,500 and 90,000 plants ha^-1^ and N treatments of 0, 180, and 360 kg N ha^-1^ in 2014 and 2015.

**FIGURE S2 |** Nitrogen use efficiency (NUE), agronomic N efficiency (AEN), and N partial productivity (PFP_N_) of summer maize for planting densities of 67,500 and 90,000 plants ha^-1^ and N treatments of 0, 180, and 360 kg N ha^-1^ in 2014 and 2015.

**FIGURE S3 |** Dry matter accumulation of summer maize at harvest for planting densities of 67,500 and 90,000 plants ha^-1^ and N treatments of 0, 180, and 360 kg N ha^-1^ in 2014 and 2015.

**FIGURE S4 |** N_2_O emission intensity and greenhouse gas intensity of summer maize for planting densities of 67,500 and 90,000 plants ha^-1^ and N treatments of 0, 180, and 360 kg N ha^-1^ in 2014 and 2015.
