# Supplementary figures and images for: Manipulating Planting Density and Nitrogen Fertilizer Application to Improve Yield and Reduce Environmental Impact in Chinese Maize Production
Source: Front Plant Sci. 2017 Jul 12;8:1234. doi: 10.3389/fpls.2017.01234 (PMC5506086; doi:10.3389/fpls.2017.01234)

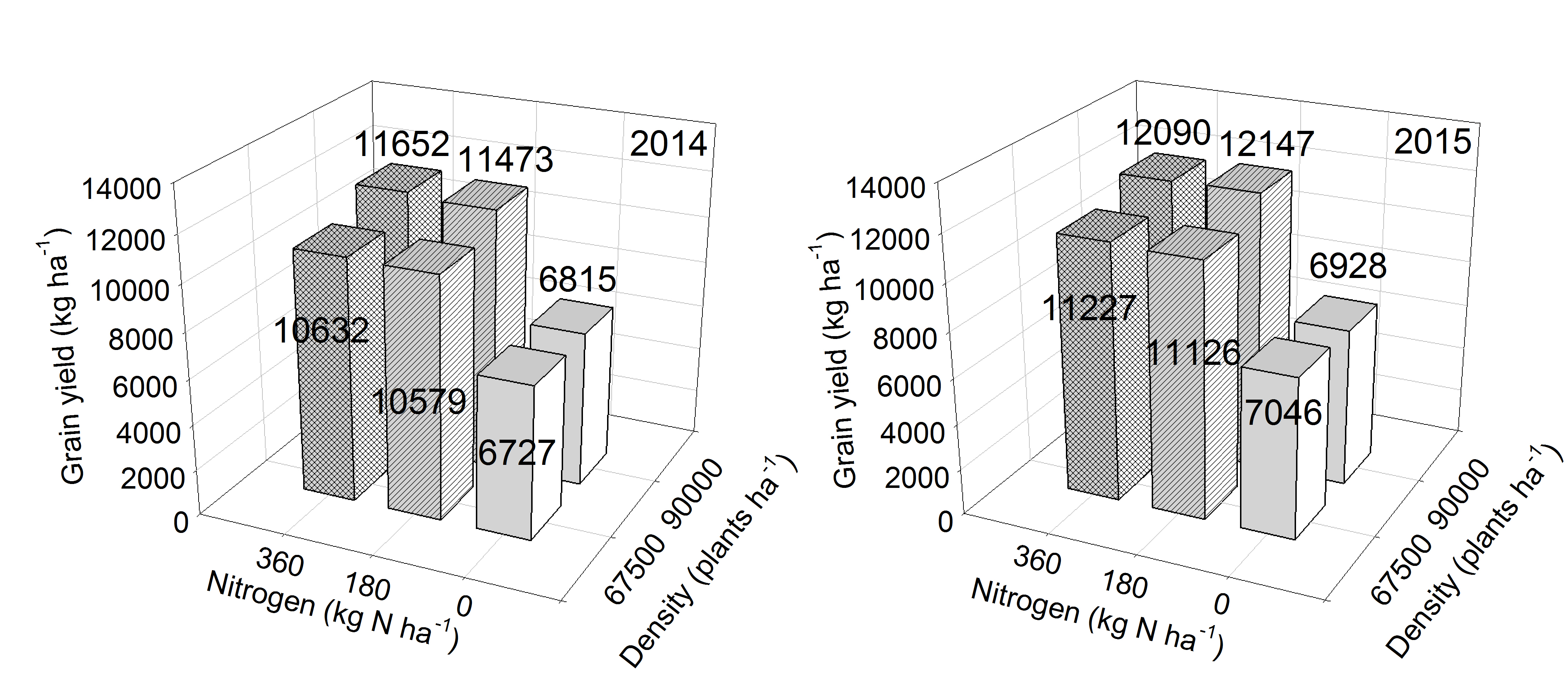

Supplement: Supplementary file 1 [file Image_1.JPEG]

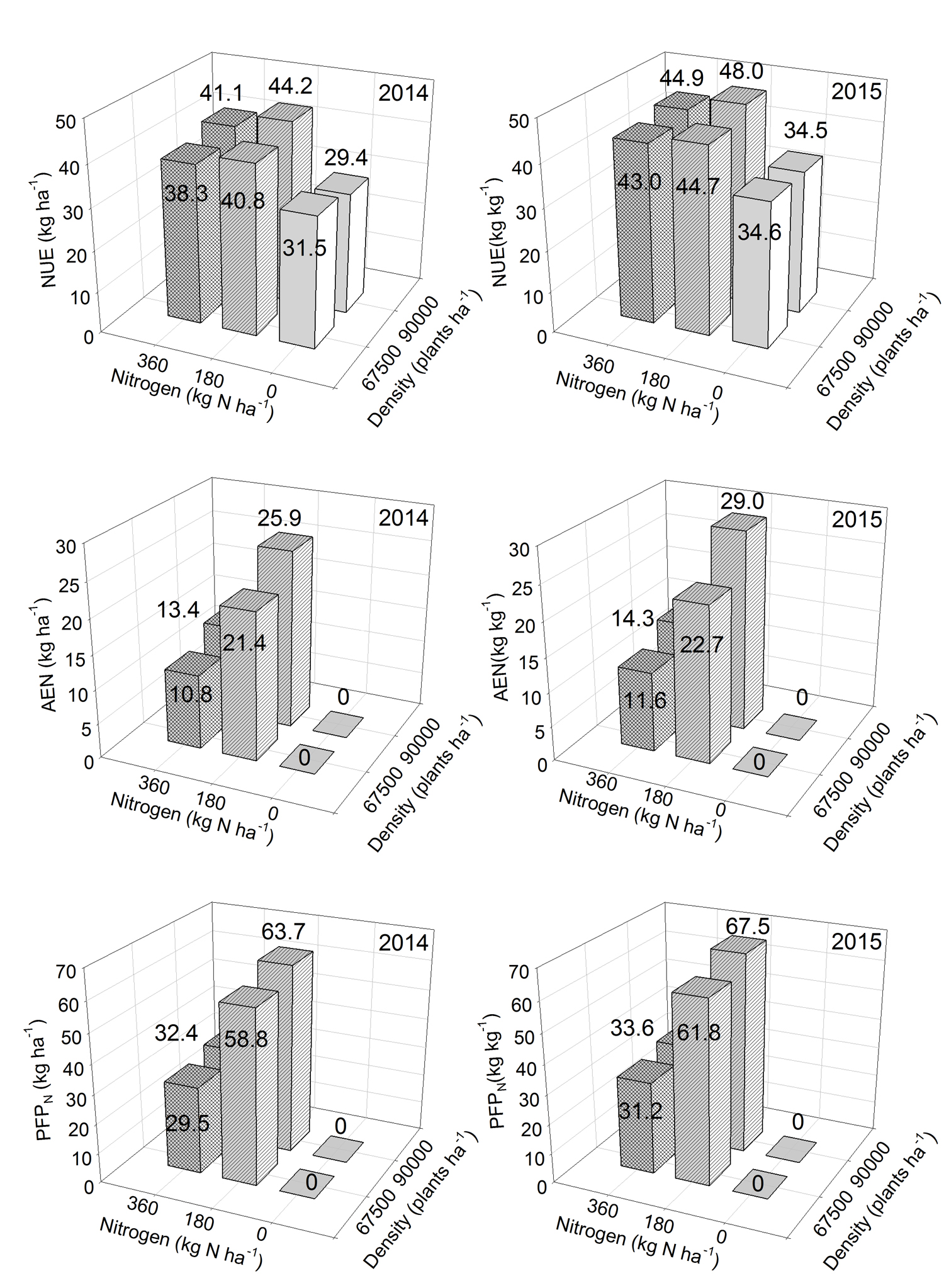

Supplement: Supplementary file 2 [file Image_2.JPEG]

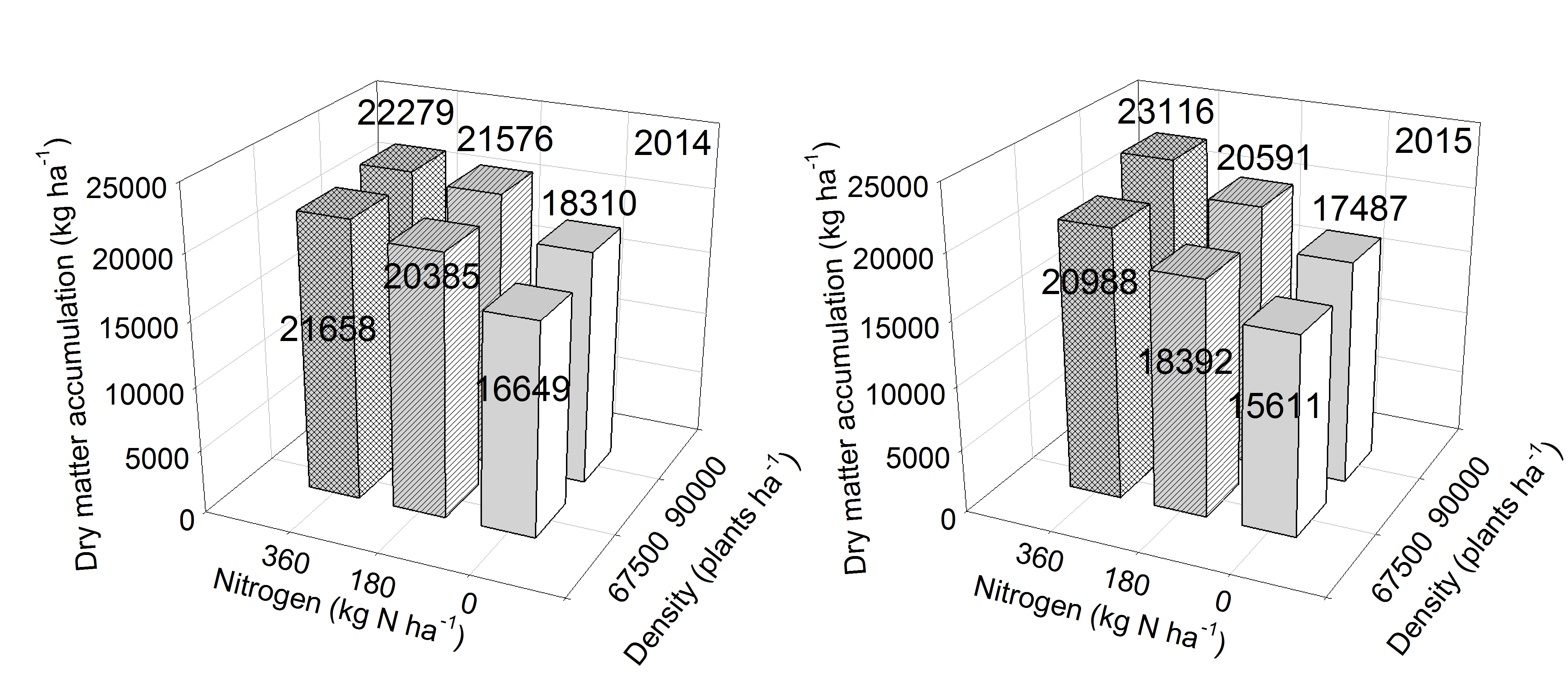

Supplement: Supplementary file 3 [file Image_3.JPEG]

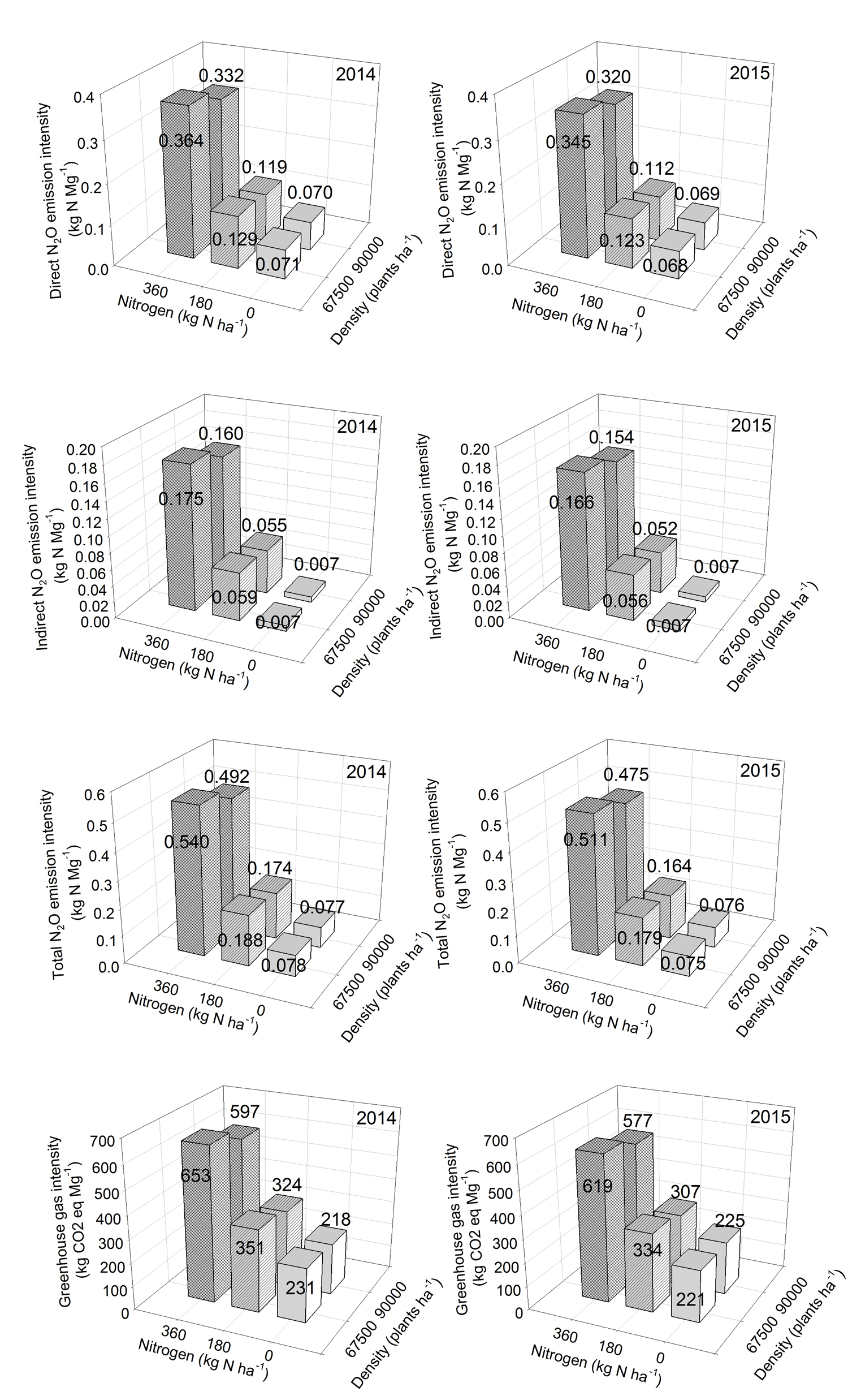

Supplement: Supplementary file 4 [file Image_4.JPEG]
